# Predischarge cerebral oxygenation and psychomotor outcome in very preterm infants: is there an association?

**DOI:** 10.1007/s00431-022-04578-6

**Published:** 2022-08-04

**Authors:** Silvia Martini, Silvia Savini, Alessandra Sansavini, Luigi Corvaglia

**Affiliations:** 1 Neonatal Intensive Care Unit, IRCCS AOU S. Orsola, Via Massarenti 13-40138, Bologna, Italy; 2 Department of Medical and Surgical Sciences, University of Bologna, Bologna, Italy; 3 Department of Psychology, University of Bologna, Bologna, Italy

**Keywords:** Near-infrared spectroscopy, Cerebral oxygenation, Preterm infants, Neurodevelopment, Psychomotor outcome, Griffiths scales

## Abstract

This observational study aimed to investigate whether predischarge cerebral oxygenation (CrSO_2_), monitored by near-infrared spectroscopy, correlates with later psychomotor outcome in very preterm infants. Infants <32 weeks’ gestation or <1500 g without evidence of major brain lesions underwent a 3-h continuous CrSO_2_ monitoring before hospital discharge. Psychomotor development was assessed at 6, 12, 18, and 24 months using the Griffiths Mental Developmental Scales. The developmental quotients (DQ) at each follow-up appointment were correlated with predischarge CrSO_2_. Significant correlations were adjusted for possible confounders. Sixty-three infants were enrolled. A significant correlation between CrSO_2_ and DQ was observed at 6 months ca (*p*=0.010), but not at later psychomotor assessments. This correlation was confirmed significant (*b*=0.274, *p*=0.038) even after the adjustment for relevant covariates.

*   Conclusion*: According to these preliminary findings, the association between predischarge CrSO_2_ and psychomotor development over the first 24 months in preterm infants without major brain lesions is time-limited. Hence, this parameter may not represent an effective predictor for medium-term neurodevelopment.

**Table:** 

**What is Known:** *• Prematurity is a major risk factor for adverse neurodevelopment. * *• The validation of clinical tools for psychomotor outcome prediction may aid to identify high-risk preterm infants who might benefit from early interventions.*	
**What is New:** *• In infants without major brain lesions, predischarge CrSO*_*2*_ *correlates with psychomotor outcome at 6 months ca but not later, indicating a short time predictability.*

**What is Known:**

*• Prematurity is a major risk factor for adverse neurodevelopment. *

*• The validation of clinical tools for psychomotor outcome prediction may aid to identify high-risk preterm infants who might benefit from early interventions.*

**What is New:**

*• In infants without major brain lesions, predischarge CrSO*_*2*_ *correlates with psychomotor outcome at 6 months ca but not later, indicating a short time predictability.*

## Introduction

Despite recent improvements in neonatal care, preterm birth is still burdened by a significant risk of neurocognitive disabilities [1]. Due to the intrinsic neuroplasticity that characterizes early brain development, a timely establishment of supportive interventions may counteract the functional constraints related to prematurity [2]. The validation of predicting tools for neurodevelopmental outcomes might help to identify preterm neonates who could benefit from these habilitative interventions. Near-infrared spectroscopy (NIRS) provides a non-invasive continuous monitoring of cerebral oxygenation (CrSO_2_). Evidence on the possible association between CrSO_2_ and neurodevelopment in preterm infants is limited to early postnatal phases [3–5], when transitional hemodynamic instability may significantly affect CrSO_2_.

We aimed to explore whether predischarge CrSO_2_, monitored in clinically stable preterm infants without evidence of major brain lesions, correlates with psychomotor outcome over the first 24 months.

## Materials and methods

Preterm infants with a gestational age (GA) <32 weeks or birth weight (BW) <1500 g admitted to the Neonatal Intensive Care Unit of S. Orsola-Malpighi University Hospital, Bologna, Italy, between February 2013 and June 2014 were consecutively enrolled in this observational study. Infants with major congenital abnormalities, hypoxic-ischemic encephalopathy, or neuroradiological evidence of major brain lesions (i.e., periventricular leukomalacia, intraventricular hemorrhage grade >II, or post-hemorrhagic ventricular dilatation) were excluded. Due to its influence on CrSO_2_, a hematocrit <30% at the time of NIRS monitoring was also considered an exclusion criterion. The study was conducted in conformity with the principles and regulations of the Helsinki Declaration. Approval was granted by the Ethics Committee of S. Orsola-Malpighi Hospital, Bologna, Italy (ref. EM 236/2016/U). Written, informed consent to participate in the study was obtained from the infants’ parents.

CrSO_2_ was monitored using an INVOS 5100 oximeter (Medtronic, Boulder, CO, USA) when the infants met the following criteria for hospital discharge [6]: physiologic stability, self-ventilating in air, stable cardiorespiratory function (i.e., no need for cardioactive drugs), full enteral feeding (160 ml/kg/day), sustained weight gain. The NIRS sensor was placed on the central forehead and maintained in the same position for the whole monitoring period. NIRS recording was started after at least 30 min of stable signal and continued for 3 h. During this period, handling procedures were minimized and feeds were administered prior to or after the monitoring. After the recording, NIRS traces were visually inspected and artifacts were removed. CrSO_2_ values were averaged over 3 h and used for statistical analysis. The coefficient of CrSO_2_ variability, which is defined as the ratio of the standard deviation (SD) to the mean of a given set of measurements and provides an estimation of the signal stability [7], was also calculated.

The following clinical data were retrieved: intrauterine growth restriction (IUGR, defined as a fetal growth reduction resulting in BW<10^th^ percentile for GA), sepsis development (defined as positive blood culture in the presence of clinical signs of infection), bronchopulmonary dysplasia (defined by the need for supplemental oxygen at 36 weeks’ postmenstrual age), and maternal education level (basic education, high school degree, university/master’s degree).

At 6-, 12-, 18-, and 24-month corrected age (CA), the enrolled infants were included in the local neurodevelopmental follow-up program performed by means of the Griffiths Mental Development Scales 0–2 years [8]. These scales investigate five developmental areas (locomotor, personal and social skills, hearing and language, eye and hand coordination, performance) and, based on the quotients for each of the aforementioned sub-scales, provide a general developmental quotient (DQ) of the infant’s abilities. The scales were administered by a trained neuropsychologist, blind to the NIRS data. The quotients were calculated using the tables of standardized scores, corrected for GA. Based on the standard deviations (SD) of DQ, psychomotor impairment was defined as mild (−1 to −2 SD), moderate (−2 to −3 SD), and severe (<−3 SD) [8].

### Statistical analysis

A sample size of 62 subjects was calculated for a two-tailed alpha of .05 and power of 80% to detect a correlation coefficient ≥0.35. Data distribution was evaluated by the Shapiro-Wilk test. Numerical variables were summarized as mean±SD or median (interquartile range) as appropriate; categorical variables were summarized as frequencies and percentages. Since both mean CrSO_2_ values and DQ scores followed a normal distribution, the correlation between CrSO_2_ and DQ at each follow-up appointment was analyzed with Pearson’s test. A hierarchical multiple regression model was built to adjust the observed results for covariates known to influence preterm infants’ neurodevelopment. Statistical analysis was performed with IBM SPSS Statistics version 25.0. The significance level was set at *p*<0.05.

## Results

Sixty-three infants were included in this study. The clinical characteristics of the study population are summarized in Table 1. The mean postmenstrual age at NIRS monitoring was 34.8±2.3 weeks. Mean predischarge CrSO_2_ was 71.1±7.2%, with a mean coefficient of variation of 0.04±0.01. Only 5 (8%) infants had mean CrSO_2_ values <55%.

**Table 1:** Characteristics of the study population

**Clinical characteristics**	***n*=63**
Gestational age (GA), weeks (mean ± standard deviation [SD])	28.7 ± 2
GA <28 weeks, *n* (%) Birth weight (BW), g (mean ± SD) BW <1000 g, *n* (%) Males, *n* (%) Twins, *n* (%) Intrauterine growth restriction, *n* (%) Vaginal delivery, *n* (%) Apgar score at 5’ (mean ± SD)	16 (25.4) 1133 ± 321 21 (33.3) 27 (43) 24 (38) 11 (17) 6 (9.5) 8 ± 1
Prematurity-related complications, *n* (%)	
Bronchopulmonary dysplasia Sepsis Retinopathy of prematurity ≥stage 3 Necrotizing enterocolitis stage ≥II	11 (17.5) 6 (9.5) 1 (1.6) 0 (0)
Maternal education level, *n* (%)	
Basic education High school degree University/master’s degree	15 (24) 32 (51) 16 (25)

The mean CA at each neurodevelopmental follow-up appointment was 6±0.3, 12.2±0.5, 18.1±0.3, and 24.2±0.4 months. The mean DQ scores at 6, 12, 18, and 24 months were 105±14.4, 99.6±9.4, 91.3±13.2, and 90.9±14.6, whereas the prevalence of moderate-to-severe psychomotor impairment was 3.6%, 0%, 11.8%, and 13.2%, respectively. At 24 months, none of the infants had developed major neurological sequelae (i.e., cerebral palsy, vision impairment, or hearing loss).

A significant association between predischarge CrSO_2_ and DQ was observed at 6 months (*r*=0.347, *p*=0.010), but not at 12 (*r*= 0.093, *p*=0.497), 18 (*r*=0.086, *p*=0.546), and 24 months CA (*r*=0.243, *p*=0.108). The positive correlation between predischarge CrSO_2_ and DQ at 6 months ca was confirmed significant (*b*=0.274, *p*=0.038) even after adjustment for GA, IUGR, sepsis, bronchopulmonary dysplasia, and maternal education level.

## Discussion

According to the present results, predischarge CrSO_2_ in preterm infants with no evidence of overt brain lesions significantly correlates with DQ at 6 months CA, but not later.

Prematurity is associated with typical changes in brain structures and connectivity, with long-term effects on cognitive, social, and language skills [9–11]; hence, predicting neurodevelopmental outcomes in the preterm population would aid to reduce the ensuing burden of disability.

To date, the role of early cerebral NIRS monitoring in predicting neurodevelopment has been investigated only in a few studies, which reported a significant association between low CrSO_2_ and adverse outcomes at 15 and 24 months CA [3–5], while in the present study, this association was limited to earlier assessments. However, several methodological differences with these studies need to be acknowledged. First, NIRS monitoring was performed in different time periods: Verhagen et al. [4] assessed CrSO_2_ on days 1 to 5, 8, and 15, while Cerbo et al. [3] and Alderliesten et al. [5] carried out a continuous CrSO_2_ monitoring over the first 48 and 72 h, respectively. During early postnatal phases, multiple conditions (e.g., patent ductus arteriosus, hypotension, mechanical ventilation) can alter preterm infants’ hemodynamics, resulting in a reduced CrSO_2_ and increasing the risk of brain injury. However, early CrSO_2_ is blind to the effect of several prematurity-related complications that can later occur during hospital stay (e.g., bronchopulmonary dysplasia, sepsis, necrotizing enterocolitis) and are known to affect preterm infants’ neurodevelopment. By monitoring CrSO_2_ before discharge in the most stable clinical conditions, we aimed to obtain a snapshot of the status of brain parenchyma net of the occurrence of previous complications. Moreover, the development of a predictive tool for psychomotor outcome in the predischarge phase may have potentially useful implications to plan taking-into-care and supportive interventions by the community services for at-risk infants.

Differently from the previous studies, we chose to exclude infants with evidence of brain lesions, which can affect CrSO_2_ measurements and are associated with adverse neurodevelopment [12]. Depending on local resource allocation policies, preterm infants without such major risk factors as overt brain damage may not be included in specific follow-up programs, with the risk of missing possible impairments; hence, a low-cost and non-invasive tool for neurodevelopment prediction in this specific population may have useful clinical implications. However, the exclusion of preterm neonates with major brain injury from the present study may have contributed to the lack of medium- and long-term predictivity of CrSO_2_ on neurodevelopment.

The following study limitations need to be acknowledged. The small number of infants with abnormal DQ did not allow to split the study population according to the degree of psychomotor impairment. Similarly, the low percentage of infants with CrSO_2_ values <55% precluded to stratify NIRS data according to this threshold adopted in early postnatal studies [4, 5]. The 3-h timescale adopted for NIRS measurements may be relatively short to provide a consistent CrSO_2_ assessment; however, the stable clinical and hemodynamic conditions in the predischarge phase and the minimal CrSO_2_ variability support the reliability of this monitoring window.

Although predischarge CrSO_2_ correlates with psychomotor development at 6-month CA, we failed to demonstrate a medium-term association, consistently with the several factors that may contribute to influence neurodevelopment at increasing ages. Given the short-term correlation observed in the present study, whether CrSO_2_ monitoring at each follow-up evaluation may be useful to predict preterm infants’ neurodevelopment from time to time, may warrant being explored.

## Data Availability

Data are available from the corresponding author upon reasonable request.

## Abbreviations

BW: Birth weight
CA: Corrected age
CrSO_2_: Cerebral oxygenation
DQ: Developmental quotient
GA: Gestational age
IUGR: Intrauterine growth restriction
NIRS: Near-infrared spectroscopy
SD: Standard deviation

## Code availability

N/A.
